# Detection of plant microRNAs in honey

**DOI:** 10.1371/journal.pone.0172981

**Published:** 2017-02-27

**Authors:** Angelo Gismondi, Gabriele Di Marco, Antonella Canini

**Affiliations:** Department of Biology, University of Rome “Tor Vergata”, via della Ricerca Scientifica 1, Rome, Italy; University of Balochistan, PAKISTAN

## Abstract

For the first time in the literature, our group has managed to demonstrate the existence of plant RNAs in honey samples. In particular, in our work, different RNA extraction procedures were performed in order to identify a purification method for nucleic acids from honey. Purity, stability and integrity of the RNA samples were evaluated by spectrophotometric, PCR and electrophoretic analyses. Among all honey RNAs, we specifically revealed the presence of both plastidial and nuclear plant transcripts: RuBisCO large subunit mRNA, maturase K messenger and 18S ribosomal RNA. Surprisingly, nine plant microRNAs (miR482b, miR156a, miR396c, miR171a, miR858, miR162a, miR159c, miR395a and miR2118a) were also detected and quantified by qPCR. In this context, a comparison between microRNA content in plant samples (i.e. flowers, nectars) and their derivative honeys was carried out. In addition, peculiar microRNA profiles were also identified in six different monofloral honeys. Finally, the same plant microRNAs were investigated in other plant food products: tea, cocoa and coffee. Since plant microRNAs introduced by diet have been recently recognized as being able to modulate the consumer’s gene expression, our research suggests that honey’s benefits for human health may be strongly correlated to the bioactivity of plant microRNAs contained in this matrix.

## Introduction

MicroRNAs (miRNAs), together with short interfering RNAs (siRNAs) and other small groups of small RNA sequences, are included in the large class of small RNAs (sRNAs). MiRNAs are non-coding RNAs, highly abundant in living cells and whose length ranges between 19 and 24 nucleotides [1–2].

The first miRNA (*lin-4*) was identified, by Lee et al. [3], in the nematode *Caenorhabditis elegans* Maupas. Since then, miRNAs have been found both in simpler organisms, such as *Chlamydomonas reinhardtii* P.A.Dang., and in superior ones, included *Homo sapiens* L., suggesting that their function was not merely an accessory but was indispensable during evolution [4].

MiRNAs are produced from endogenous genes (MIR genes) which are phylogenetically conserved and which may be evolved independently within each life kingdoms [5]. Indeed, the existence of the same miRNA in organisms belonging to different kingdom (i.e. miR854 detected in *Arabidopsis thaliana* (L.) Heynh., *C*. *elegans*, *Mus musculus* L. and *H*. *sapiens*) remains scarcely documented [4].

MiRNAs have been demonstrated to be fundamental for the modulation of gene expression in the cells where they are synthesized. For example, in humans, miRNAs are able to regulate about 30% of the whole gene set [6]. In particular, since their production has been reported to be restricted to specific temporal phases and strongly dependent on cell tissue, various studies have suggested miRNA involvement in several biological processes, such as development, differentiation, proliferation, response to biotic and abiotic stresses and the establishment of pathological states (i.e. tumor) [7–9].

In general, each miRNA determines the gene silencing of one, or more, specific RNA messengers (mRNAs). For miRNAs, two action mechanisms have been widely illustrated, although some minor aspects still remain ambiguous. When miRNA is perfectly (or almost) complementary with a portion of nucleotide sequence of its mRNA target (usually 3'-UTR in animal cells and 3'-UTR, 5'-UTR or even a motif present inside an ORF in plant systems), the transcript is rapidly degraded, probably favoring the removal of the poly-A tail, thus making it less stable. *Vice versa*, if the pairing between miRNA and gene messenger is imperfect, mRNA is subjected to translation repression, possibly altering ribosome sliding or stability on the transcript or inhibiting the coupling of eIF4E factor with mRNA cap [9, 10].

MiRNA biogenesis has been widely described in the literature and follows a very similar pathway in both animal and plants, although some steps are carried out by orthologue enzymes. In briefly, from the MIR genes, miRNAs are transcribed by RNA polymerase II and then finely processed in the nucleus before being transported into the cytoplasm. Here, miRNAs can perform their regulatory activity on mRNA targets by being associated to the RISC complex (RNA induced silencing complex) [9, 11, 12].

The discovery of the existence of cross-kingdom interactions mediated by miRNAs has been one of the most significant breakthroughs of the last decade. However, only a few studies have reported this *phenomenon* [13–16]. Among them, probably the most important is that of Zhang et al. [17], where the authors demonstrated that *Oryza sativa* L. miRNA186a could be detected in both the blood and tissues of mammals on a rice diet. Moreover, this plant miRNA, surprisingly still in active form, showing sequence homology with the human mRNA codifying for Low Density Lipoprotein Receptor Adapter Protein 1 (LDLRAP1), was also able to reduce the translational process of LDLRAP1 transcript in animal cells, thus determining an increase of LDL level in plasma. These *data* clearly confirmed that plant miRNAs, introduced by diet, could strongly influence consumers’ gene expression, acting as communication molecules between different kingdoms. However, this potential role of plant miRNAs is strongly dependent on the fact that, with respect to animal ones, they are characterized by a 3’-terminal nucleotide whose sugar is methylated in position 2’: this feature endows the miRNAs with extreme stability, resistance to degradation (i.e. low pH), preservation to temperature variation (i.e. boiling) and absorption processes by intestinal *mucosae* [17–21]. All this evidence suggests that the beneficial effects of a plant food-based diet may be correlated to the bioactivity of plant miRNAs.

Therefore, the main objective of this research has been to demonstrate, for the first time in literature, the existence of plant RNAs and miRNAs in different samples of monofloral honeys (*Malus domestica* Borkh., *Robinia pseudoacacia* L., *Castanea sativa* Mill., *Eucalyptus* sp., *Tilia cordata* Mill., *Rosmarinus officinalis* L.). In fact, since honey derives from the re-elaboration of floral secretions by honeybees (*Apis mellifera ligustica* Spinola) [22], we had initially hypothesized that plant RNAs and miRNAs collected in nectar could be directly transferred into its derivative honey. During our investigations, several RNA extraction methods were followed and compared. The detection of plant miRNAs in honey could explain, at least in part, the biological properties associated with this natural matrix [23, 24]; it would also suggest new possible potential medical applications for this food.

Finally, other plant derived-commercial products, such as desiccated tea leaves (*Camellia sinensis* L.) and cocoa (*Theobroma cacao* L.) and coffee seed powders (*Coffea arabica* L.), have been similarly analyzed for analogous purposes.

## Materials and methods

### Plant materials and honey samples

Forty *M*. *domestica* flowers were sampled from 4 adult apple trees (ten flowers per tree) grown and cultivated in the Botanical Gardens of Rome “Tor Vergata”. *R*. *pseudoacacia* nectar (about 4 mL) was collected, by a Hamilton syringe (max vol. 50 μL), from Black Locust flowers harvested, in the Botanical Gardens of Rome “Tor Vergata”, from 4 different bloomed trees. Apple flowers and *R*. *pseudoacacia* nectar were instantaneously processed after sampling. Honeys were kindly provided by the Honey Research Center of the University of Rome “Tor Vergata”, after certification by melissopalynological analysis [22]. In particular, 3 different samples for each typology of monofloral honey (*M*. *domestica*, *R*. *pseudoacacia*, *C*. *sativa*, *Eucalyptus* sp., *T*. *cordata*, *R*. *officinalis*) were studied. Desiccated tea leaves (*C*. *sinensis*) and cocoa (*T*. *cacao*) and coffee seed powders (*C*. *arabica*) were obtained each from 3 respective commercial sachets; as such, they were industrially processed and ready to be used by consumers.

### RNA extraction procedures and spectrophotometric quantification

For RNA extraction, plant samples (flowers, leaves, seed powders) were completely pulverised using mortar and pestle, in the presence of liquid nitrogen, while honeys and nectars were used in their original form. Three different extraction procedures were employed. According to the first method, Pure Link RNA Kit (Ambion, Life Technologies; *total RNA extraction kit*), samples were lysed in the presence of guanidinium isothiocyanate, a chaotropic salt able to protect RNA from endogenous RNases; the samples were then processed through a Spin Cartridge containing a clear silica-based membrane that bound RNA. Impurities were removed by subsequent washing, and purified total RNA (long and short sequences) was eluted in RNase-free water. By using mirPremier microRNA Isolation Kit (Sigma-Aldrich; named *miRNA extraction kit*), samples were treated by a 2-mercaptoethanol enriched-lysis buffer that specifically solubilized small/micro RNA, inactivated ribonucleases and neutralized interfering plant secondary metabolites. Large RNA and genomic DNA, which both remained insoluble, were removed by centrifugation from the lysate, along with other cellular debris. Therefore, only small/micro RNA was captured onto a silica binding column, as specified in the manufacturer’s guidelines. Residual impurities were removed by repeated washings, and small/micro RNA was eluted in RNase-free water. The third extraction method (named *home-made RNA extraction*) was carried out according to a Sambrook [25] laboratory manual method modified as follows: samples were incubated, for 1 h at 37°C, with lysis buffer (0.1 M tris HCl pH 7.5; 0.1 M NaCl; 0.01 M EDTA; 2% SDS; 1 mg/mL proteinase K; 5% polivinilpirrolidone; 5% cetyl trimethylammonium bromide). Then, 0.1 volumes of 2 M sodium acetate pH 4 and 1 volume of phenol/chloroform pH 4.5 were added to the solutions. Each sample was vortexed for 5 minutes and centrifuged at 13,000 RPM speed for another 5 minutes. Supernatant was collected and subjected to three further extractions, two with phenol/chloroform and one with only cholorofom. Subsequently, purified supernatant was supplemented with 0.7 volumes of 100% isopropanol, in order to precipitate DNA which was then removed by a sterile *pasteur* pipette. The whole solution was conserved at -70°C for 1h and, then, centrifuged for 30 minutes at 4°C at 13,000 RPM. Finally, the pellet was resuspended in RNase-free water and treated, according to manufacturer’s guidelines, with DNAse I (Promega, Italy), in order to destroy residual contaminating DNA molecules. All RNA samples were stored at -70°C until their analysis. RNA concentration and purity were determined with a Nanodrop ND1000 spectrophotometer (NanoDrop Technologies). RNA separation and visualization was performed by agarose-formaldehyde gel electrophoresis according to the Sambrook [25] laboratory manual.

### cDNA retrotranscription, PCR and real-time PCR (qPCR) analyses

Total cDNA was synthesized by joining, in a final volume of 25 μL for 90 min at 37°C, the following reagents: 0.4 mM each dNTP (Euroclone, Milan—Italy), 20 U ribolock RNase inhibitor (Thermo Scientific, Milan—Italy), 0.5 μg random hexamer primers (Invitrogen, Milan—Italy), 200 U moloney murine leukemia virus reverse transcriptase (Promega, Italy), 1X enzyme buffer, 10 mM dithiothreitol and 0.5 μg RNA, previously heated at 65°C for 2 minutes. By contrast, the synthesis of microRNA cDNAs was carried out by using a reverse transcription kit specific for miRNAs (miRCURY LNA Universal RT microRNA PCR, Synthesis Kit II; EXIQON), according to manufacturer’s instructions. In particular, 10^8^ copies of a synthetic spike-in control miRNA (UniSp6, EXIQON) were added for each retrotranscription reaction, in order to control the absence of nucleases during the procedures and the efficiency of cDNA synthesis and qPCR assay. PCR amplifications of plant genes (ribulose 1,5-bisphosphate carboxylase/oxygenase large subunit, rbcL; maturase K, matK; 18S nuclear ribosomal DNA, 18S rDNA) were performed by a Biorad (IQ5) thermocycler. PCR procedure and conditions, reagent concentrations and amplicon detection through gel electrophoresis were all identical to those widely reported in Gismondi et al. [26]. The choice of primer sequences and relative annealing temperatures were based on Gismondi et al. [27], for rbcL (F1-R2; 201 base-pairs/bp) and matK (F1-R2; 217 bp), and Gismondi et al. [28], for 18S rDNA (F-R; 844 bp). Real time PCR, for the quantification of each miRNA, was performed in a 10 μL reaction volume containing: 10 ng cDNA, 50% SYBR green (Kapa SYBR Fast qPCR kit; Kapa Biosystems, Woburn, MA, USA) and 1 μL of the mixture presenting both miRNA specific PCR primers (microRNA LNA PCR primer sets, EXIQON). As reported on EXIQON instruction manual, amplification was performed using a Biorad (IQ5) thermocycler with the following parameters: (a) initial denaturation at 95°C for 10 min; (b) 45 cycles of denaturation at 95°C for 10 seconds (sec), primer annealing temperature at 60°C for 1 minute and extension at 60°C for 30 sec; (c) production of disassociation curve from 50 to 90°C (rate: 1.6°C every sec) for the verification of the results. qPCR analysis was carried out on the following miRNAs [29, 30]: miR482b (5’-UCUUUCCUAUCCCUCCCAUUCC-3’), miR156a (5’-UGACAGAAGAGAGUGAGCAC-3’), miR396c (5’-UUCCACAGCUUUCUUGAACUU-3’), miR171a (5’-UGAUUGAGCCGCGCCAAUAUC-3’), miR858 (5’-UUCGUUGUCUGUUCGACCUGA-3’), miR162a (5’-UCGAUAAACCUCUGCAUCCAG-3’), miR159c (5’-GAAUUCCUUCUCCUCUCCUUU-3’), miR395a (5’-CUGAAGUGUUUGGGGGAACUC-3’) and miR2118a (5’-CUACCGAUGCCACUAAGUCCCA-3’). The 5S rRNA (whose specific primers were developed and designed by EXIQON Service on the basis of plant 5S rDNA sequences, including *A*. *thaliana* [GenBank: AB073495.1]) was used as internal loading control to normalize of qPCR results (relative quantization). In fact, the amount of each miRNA was determined using the 2^-ΔΔCt^ formula, where the threshold cycle (Ct) of the target miRNA measured in one sample is normalized with respect to the internal reference gene (5S rRNA, ΔCt) and to the respective value obtained in the second sample (ΔΔCt). qPCR negative controls (without template, Neg. CNT1; without primers, Neg. CNT2) were performed throughout to confirm adequacy and accuracy of the detection system. Results were expressed as mean ± standard deviation (s.d.) and all the experiments were repeated at least three times starting from different and independent RNA extractions. The significance of the results was measured by one-way ANOVA test using PAST software (p values < 0.05 were considered significant).

## Results and discussion

The beneficial effects of a plant food-based diet on human health have been widely documented in literature [31,32]. In general, such epidemiological evidence has been associated with the bioactivity of plant secondary metabolites (i.e. simple phenols, flavonoids) [33]. However, the bioavailability of these molecules, being very limited [34], might not always justify the positive influence of such type of alimentation. On the other hand, it has been recently demonstrated that plant miRNAs were able to modulate cell gene expression of animals absorbing them by diet [17]. This extraordinary result supports the hypothesis that medicinal and food plants might finely regulate human metabolism and physiology by their miRNA content, not only through specific chemical compounds.

For these reasons, the present work aimed to investigate if plant miRNAs could be detectable even in the honey, a sugary matrix which derives from the re-elaboration of plant products (i.e. floral nectar, extrafloral secretions) by honeybees (*Apis mellifera ligustica* Spinola) [22], in order to justify and improve scientific knowledge about the biological activities associated with this natural food [23, 24].

To determine the best procedure for miRNA extraction, three different methods were carried out, named respectively *total RNA extraction kit*, *home-made RNA extraction* and *miRNA extraction kit*, as widely reported in “Materials and Methods” section.

The first sample we decided to process was an apple honey because, given the recent publication of a paper describing *M*. *domestica* miRNome and which we used as our main reference for the choice of miRNAs to detect in the current study [29]. To monitor the efficiency of the protocols, since apple honey originates from apple floral nectar, *M*. *domestica* flowers were also subjected to miRNA extraction.

As shown in Table 1, for each extraction method, we have reported the yield of RNA (in ng) per mg of sample (apple flower or honey) and the relative ratios of spectrophotometric absorbances at 230, 260 and 280 nm. In fact, the ratios A_260/280_ and A_260/230_ are usually employed to measure the purity level of nucleic acids since nucleotides, RNA and DNA all absorb at 260 nm; A_260/280_ and A_260/230_ ratios of, respectively, ~2.00 and ~2.00–2.20 generally indicate a “pure” RNA sample [35].

**Table 1:** Comparison of RNA extraction methods (total RNA extraction kit, home-made RNA extraction and miRNA extraction kit) from apple flowers and honeys.

**Total RNA extraction kit**			
*sample*	*ng RNA/mg sample*	*A*_*260/280*_	*A*_*260/230*_
Apple flower	48.00 ± 2.16	1.67 ± 0.05	0.71 ± 0.03
Apple honey	1.42 ± 0.04	1.63 ± 0.07	0.30 ± 0.01
**Home-made RNA extraction**			
*sample*	*ng RNA/mg sample*	*A*_*260/280*_	*A*_*260/230*_
Apple flower	848.70 ± 32.88	1.47 ± 0.03	1.86 ± 0.05
Apple honey	24.75 ± 1.01	1.35 ± 0.06	1.57 ± 0.03
**miRNA extraction kit**			
*sample*	*ng RNA/mg sample*	*A*_*260/280*_	*A*_*260/230*_
Apple flower	24.96 ± 1.18	1.87 ± 0.06	2.03 ± 0.04
Apple honey	0.68 ± 0.02	1.91 ± 0.04	2.12 ± 0.07

All values are reported as mean ± s.d. of three independent measurements

*Home-made RNA extraction* recovered the highest concentrations of RNA, in all samples, compared to the other two methods. It was unexpected to notice that *total RNA extraction method* was not able to purify an amount of RNA similar to that obtained by *home-made RNA extraction*, while the same result was quite predictable for *miRNA extraction kit* which is specifically designed to extract only small and micro RNAs (see “Materials and Methods” section). Surprisingly, the spectrophotometric analyses revealed the presence of RNAs even in apple honey samples, demonstrating that these molecules were directly transferred and preserved from their original sources to the final product. However, in all cases, the amount of RNA extracted from plant tissue was about 35-fold higher than from honey, indicating a strong degradation activity of the RNA during honey production.

RNA collected by *total RNA extraction method*, presenting an A_260/280_ ratio between 1.50 and 1.60 (Table 1), did not prove to be completely pure, although a silica-based membrane had completely purified the extract. Moreover, very low values of A_260/230_ suggested that both flower and honey samples could have been contaminated by carbohydrates and plant secondary metabolites that strongly absorb visible light at, or near, 230 nm [36]. These data may be justified by the fact that this extraction kit, although designed for a wide range of matrixes (i.e. animal cells, blood, fungi), does not include a specific step able to separate RNA from sugars and other plant compounds. Indeed, apple honey, being rich of fructose and glucose, revealed an extremely small A_260/230_ ratio (0.30).

*Home-made extraction*, as in the case of the previous method, has allowed us to obtain an impure RNA: maybe, the presence of proteins, derived from the samples, or phenol residues, used during the extraction procedure, negatively affected the A_260/280_ ratio [36]. After all, the absence of a specific RNA purification filter, as that applied in the previous protocol, could justify this phenomenon. On the other hand, the application of detergents, such as CTAB and PVP, strongly reduced the contamination levels of carbohydrates and plant compounds in the final eluate, as suggested by the A_260/230_ ratios reported in Table 1. However, apple honey A_260/230_ value still revealed the predominant sugary nature of this matrix.

The best A_260/280_ and A_260/230_ ratios were measured for the small/micro RNAs purified by *miRNA extraction kit*. In both samples, no peculiar contamination was detected, while even elevated purity values reached (Table 1).

According to this evidence, we have concluded that *miRNA extraction kit*, could be the most suitable system for extracting microRNAs from honey, followed by the *home-made RNA extraction* method.

**Fig 1:**
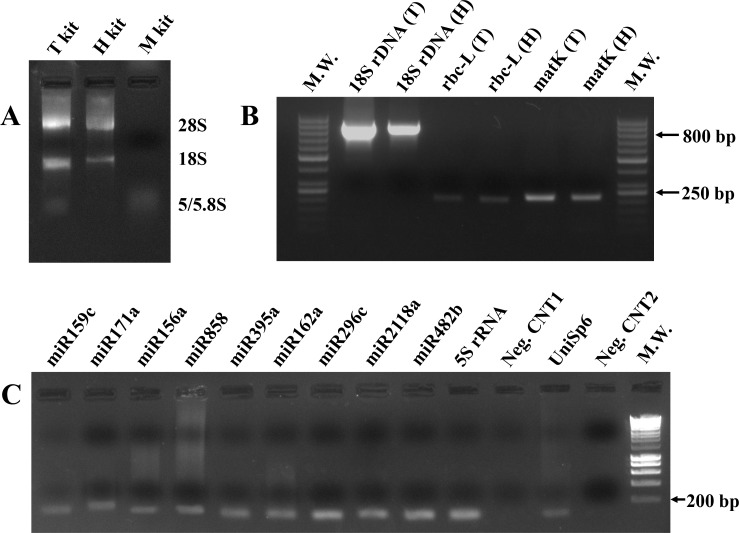
Nucleic acid gel electrophoresis and visualization. **(A)** A representative image (of all the others produced with similar results) of RNAs purified from apple flowers by three different extraction methods, *total RNA extraction kit* (T kit), *home-made RNA extraction* (H kit) and *miRNA extraction kit* (M kit), and separated on formaldehyde-agarose gel was shown. Large (i.e. 28S and 18S rRNAs) and small/micro (i.e. 5/5.8S rRNAs) RNA bands were easily detectable under UV light. **(B)** Here, the example of one of the various independent agarose gel electrophoreses performed, with similar results, was reported. In particular, amplification products obtained in PCRs containing cDNA synthesized from honey RNA extracted by *total RNA extraction kit* (T) and *home-made RNA extraction* (H) and specific primers for 18S rDNA, rbc-L and matK genes were shown. A molecular weight marker (M.W.) was also loaded in the gel to confirm the dimension in base pairs (bp) of DNA fragments. **(C)** All qPCR amplifications were loaded on agarose gel, separated and visualized to confirm the efficiency of the revealing system. The image represents one of the real-time PCR analysis performed on a honey sample subjected to *miRNA extraction kit*. Positive and negative controls (respectively named UniSp6 and Neg. CNT1/2) were carried out at everyPCR amplification and verified on gel. A molecular weight marker (M.W.) was also loaded into gel to validate the dimension in base pairs (bp) of DNA fragment signals.

In order to demonstrate the validity of the previous exctraction methods, the presence of RNA purified from apple flowers samples through the different protocols was validated by agarose-formaldehyde gel electrophoresis (Fig 1A). Large (28S and 18S) ribosomal RNAs (rRNAs) were detectable in the extracts obtained by *total RNA extraction method* and *home-made RNA extraction*, while the signal of the small and micro RNAs, including 5.8S and 5S rRNAs, was weakly visible only in the first of these samples. Moreover, in both lanes, the smears visualized above 28S rRNA band could be directly due to the presence of contaminants in these extracts, as previously revealed and suggested by their altered A_260/280_ and A_260/230_ ratios.

*Vice versa*, no band of large dimensions were observable in the lane relative to *miRNA extraction kit*. Indeed, as expected on the basis of this kit’s specific potentialities, this sample exclusively showed a low molecular weight spot, this representing all honey small and micro RNAs.

To verify stability and integrity of the RNA extracted from apple flowers and honeys with the three purification procedures, a classic reaction of retrotranscription was carried out. Consequently, each cDNA sample was used as a template for PCR amplifications of three different plant gene regions, two short sequences arranged on the plastidial genome (rbc-L of 201 bp and matK of 217 bp) and a long trait located on the nuclear one (18S rDNA of 844 bp). These regions were chosen for two principal reasons: i) They represented some of the best plant DNA barcodes, nucleotide successions highly conserved in plant kingdom and were very easy to amplify and detect [28, 37–39]; ii) They presented different genetic origin (plastidial or nuclear) and length (in bp), features which could be informative and useful for discriminating between the type of RNA molecules purified during the extraction protocols.

All PCRs performed with cDNA synthesized starting from apple honey RNA extracted by both *total RNA extraction method* and *home-made RNA extraction* produced amplicons. These fragments were detected, analyzed and verified in length, through a molecular weight (M.W.), by agarose gel electrophoresis (Fig 1B). As expected, no amplification was generated in PCR reactions containing apple honey RNA purified with *miRNA extraction kit*, suggesting how this procedure was not able to extract large/medium length RNAs. The same results were obtained from flower samples.

Our results clearly demonstrated, for the first time, the existence of plant RNAs in honey samples. Moreover, these nucleic acids, directly derived from flower nectar, appeared to be still stable, intact, undamaged and, probably, even functional within this sugary matrix.

All these *data* strongly suggested the hypothesis that, among plant RNAs, microRNAs could also exist in honey. To prove this, RNA samples, obtained by the three extraction procedures, were firstly subjected to a retrotranscription reaction specific for small and micro RNAs (miRCURY LNA Universal RT microRNA PCR, Synthesis Kit II; EXIQON) and then analyzed by real-time PCR. The presence of nine different plant miRNAs (miR482b, miR156a, miR396c, miR171a, miR858, miR162a, miR159c, miR395a and miR2118a), chosen for their abundant expression, wide distribution in various plant districts and elevated nucleotidic conservation in plant kingdom [29, 30, 40], was detected, studied and quantified with respect to plant 5S rRNA which was used as internal loading control as suggested in literature [41, 42]. In order to verify qPCR results, after amplification reaction, all samples were separated on agarose gels by electrophoresis and visualized, under UV light, by ethidium bromide staining. One of these gels, showing the short nucleotide fragments derived from miRNA real-time PCR amplifications, was reported in Fig 1C, as representative one. Positive and negative controls (respectively named UniSp6 and Neg. CNT1/2, see “Materials and Methods” section) were performed throughout to confirm adequacy, accuracy and efficiency of the qPCR revealing system.

As described in Table 1 (A, B and C panels), plant miRNAs were identified in apple honeys and quantified by qPCR, with respect to the correspondent levels detected in *M*. *domestica* flower samples. Apple honey miRNAs purified by *total RNA extraction method* (Table 2, panel A) appeared to be much less concentrated (between 10^−2^ and 10^−6^ folds) than in apple flowers.

**Table 2:** Plant miRNA detection and quantitation by qPCR in different plant-derived samples (flowers, honeys, seed powders, dried leaves).

Extraction method and samples	Plant microRNA								
	miR159c	miR171a	miR156a	miR858	miR395a	miR162a	miR396c	miR2118a	miR482b
***PANEL A*. *Total RNA extraction kit***									
*M*. *domestica* flower*	100	100	100	100	100	100	100	100	100
*M*. *domestica* honey	0.01	0.61	1.05 e^-4^	6.42 e^-4^	2. 24 e^-6^	1.73 e^-5^	9.54 e^-5^	2.10 e^-6^	2.03 e^-5^
***PANEL B*. *Home-made RNA extraction***									
*M*. *domestica* flower*	n.d.	n.d.	100	n.d.	100	n.d.	100	100	n.d.
*M*. *domestica* honey	0.02	n.d.	n.d.	n.d.	n.d.	n.d.	n.d.	n.d.	n.d.
***PANEL C*. *miRNA extraction kit***									
*M*. *domestica* flower*	100	100	100	100	100	100	100	100	100
*M*. *domestica* honey	0.17	8.08	3.90	0.51	0.77	123.97	14.46	76.84	3.04
***PANEL D*. *miRNA extraction kit***									
*R*. *pseudoacacia* nectar*	100	100	100	100	100	100	100	100	100
*R*. *pseudoacacia* honey	2.15	36.10	0.11	24.32	19.61	0.23	1.36	0.01	4.24
***PANEL E*. *miRNA extraction kit***									
*M*. *domestica* honey*	100	100	100	100	100	100	100	100	100
*R*. *pseudoacacia* honey	6955.10	158.01	29.12	5610.28	48103.56	516.94	251.40	0.074	229.74
*C*. *sativa* honey	143815.2	1139.24	11697.04	80900.23	77068.63	3779.18	10325.01	33.22	668.07
*Eucalyptus sp*. honey	839.77	67.83	89.50	6579.93	3939.66	231.34	208.49	10.51	139.47
*T*. *cordata* honey	20365.73	309.51	198.62	19808.83	5688.59	111.73	303.14	43.83	606.29
*R*. *officinalis* honey	6268.29	168.18	12.67	5381.74	12109.54	502.80	1837.92	12.41	1100.43
***PANEL F*. *miRNA extraction kit***									
*C*. *arabica* seed powder*	100	100	100	100	100	100	100	100	100
*C*. *sinensis* dried leaf	0.02	1.91	0.03	1.04	0.01	3312.85	1.36 e^-13^	0.63	0.05
*T*. *cacao* seed powder	6.08	506.30	0.32	3885.42	0.02	5784882	2.54 e^-10^	0.46	0.19

* control sample used as unit (100%)

Dark or light grey boxes correspond, respectively, to higher or lower concentrations of miRNA compared to the control

n.d. not detected

All values is reported as mean of three independent measurements; s.d.<8% of the mean value; p<0.04 vs control

According to *home-made RNA extraction* (Table 2, panel B), *M*. *domestica* honeys appeared to be completely lacking in plant small/micro RNAs, except in miR159c and 5S rRNA. In the same context, even our flower samples showed no presence of the following plant miRNAs, miR159c, miR171a, miR858, miR162a and miR482b. This evidence, in contrast with that previously obtained (Table 2, panel A), suggested that *home-made RNA extraction* was not adequate for purification of small and micro RNAs from plant tissues and their derivatives. Probably, one of the extraction steps of this protocol determined the loss of miRNA fraction, as supported by the absence of small/micro RNA signal for this sample in Fig 1A.

qPCR assay performed on RNA purified by *miRNA extraction kit* (Table 2, panel C) corroborated preceding *data* (Table 2, panel A), that is, the existence of miRNAs in *M*. *domestica* honey samples. In particular, in this case, the levels of honey miRNAs were much higher than those detected during the analysis carried out starting from RNA collected by *total RNA extraction method* (Table 2, panel A). This clearly demonstrated that *miRNA extraction kit* was the best method for miRNA purification from honey samples, in comparison to *total RNA extraction method* which underestimated our results (note how, in Fig 1A, small/micro RNA band in T kit lane was less strong than the M kit one).

Generally, we observed that plant miRNAs presented a lower concentration in apple honeys than in *M*. *domestica* flowers, suggesting degradation and/or dilution processes of miRNAs during honey production. Exceptionally, miR162a appeared to be more abundant in honey than in plant tissue (Table 2, panel C), supporting the hypothesis that this specific miRNA was more stable compared to others or accumulated in the sugary matrix from multiple nectars sampled by honeybees.

A similar analysis was also performed on *R*. *pseudoacacia* nectar and its derivative honey, starting from RNA purified by *miRNA extraction kit* (Table 2, panel D), demonstrating that plant miRNAs might be directly transferred from plant exudates in honey through honeybee activity.

Finally, a comparison of plant miRNA content among different typologies of monofloral honeys was carried out (Table 2, panel E). *C*. *sativa* honeys showed the richest miRNA profiles of all, followed by those of *T*. *cordata*, *R*. *officinalis*, *R*. *pseudoacacia* and *Eucalyptus sp*., respectively. By contrast, *M*. *domestica* honeys appeared to possess the lowest concentrations of plant miRNAs with respect to the other samples, except for miR156a and miR2118a which were higher. However, this result was hardly unexpected; in fact, as reported in Xia et al. [29], miR2118a, also known as miRC3, is one of the plant miRNAs peculiarly abundant in *M*. *domestica* tissues, especially in leaves and flowers.

In conclusion, our research clearly demonstrated that honeybees are able to collect plant RNAs together with nectar, during their forages, and to concentrate them in the honey. Of special interest is how these nucleic acids were able to preserve their integrity and stability inside the honey. However, the high concentrations of sugars in the honey and the stable conditions of the hive might justify the previous observations.

Among honey plant RNAs, microRNAs represent the most important class on account of their potential bioactivity. In fact, these molecules, if introduced by diet, could modulate the cell gene expression of all honey consumers, as recently demonstrated for rice miRNAs [17]. The chemical stability of plant miRNAs [18–21] strongly supports the hypothesis that honey miRNAs, after their assumption, are also absorbed into the blood and distributed in body tissues to perform their biological function, namely the regulation of human target mRNA translational process. This supposition would explain some of the medicinal properties of this natural matrix [23, 24] and would also suggest new putative biological applications for this food.

Plant miRNAs, ingested by humans with honey, could show affinity for mammalian mRNAs which are not normally regulated by animal miRNA or which change and alter the biological effect of endogenous miRNAs, acting on the same, similar or opposite targets. Concerning the latter hypothesis, it has indeed been widely documented that miRNA bioactivity is strongly dependent on its cell concentration and copy number of the relative target mRNA which, in turn, is finely regulated by physiological conditions, external environmental *stimuli* and internal signaling pathways [43]. According to this observation, it appears clear that even low doses of plant miRNA, as detected in some honeys, may exert their biological function on mammalian gene expression.

This study opens up new perspectives on honey’s potentially beneficial effects and strongly suggests, as a future goal, the importance of performing next generation sequencing (NGS) analyses to fully characterize the honey miRNome. At this point, it is necessary to underline how our study demonstrated that honey’s botanical and geographical origin might influence, both the quality and the quantity, of honey’s miRNA content, so suggesting the existence of a specific miRNA-based fingerprint for each honey. In addition, we cannot exclude that honey can also contain some *A*. *mellifera* miRNAs which honeybees directly release in this matrix during its production and storage in the hive.

Finally, as supplementary study, we investigated the presence of plant miRNAs in *C*. *arabica*, *C*. *sinensis* and *T*. *cacao* commercial products (Table 2, panel F); this was to verify if these molecules remained intact from the plant source to their final processed form, as in the case of honey. Coffee and cocoa seed powders showed a richer miRNA profile than did dried tea leaves. These results raise the interesting question as to whether the plant miRNAs identified in the samples above could explain the well-known antioxidant, stimulating and health properties of beverages derived from the same plants.
